# Supplementary material for: Prevalence and determinants of depression, anxiety, and perceived stress among patients with chronic comorbidity attending outpatient clinics in Addis Ababa during the COVID-19 pandemic
Source: PLOS Glob Public Health. 2026 Feb 25;6(2):e0005838. doi: 10.1371/journal.pgph.0005838 (PMC12935201; doi:10.1371/journal.pgph.0005838)
Supplement: Attachment — Submitted filename: 3. Response to Reviewers.docx [file pgph.0005838.s005.docx]

**25 Dec 2025GC**

**PLOS Global Public Health**

**Manuscript ID:** PGPH-D-25-03264

Title of paper: *Prevalence and Determinants of Depression, Anxiety, and Perceived Stress Among Patients with Chronic Comorbidities Attending Outpatient Clinics in Addis Ababa During the COVID-19 Pandemic*

**Subject: Response to the** **editor and reviewer on our manuscript**

Dear Theingi Maung Maung, Ph.D., Academic Editor, and Reviewers,

We sincerely thank the Academic Editor and the reviewers for their careful evaluation of our manuscript and for the constructive comments provided. We greatly appreciate the opportunity to revise our manuscript. We have carefully addressed all editorial and reviewer comments and believe that these revisions have significantly improved the quality and clarity of the manuscript.

All changes have been incorporated into the revised manuscript. A clean revised manuscript has been submitted, along with a separate tracked-changes version highlighting all revisions, provided as a supplemental file entitled *“Revised Manuscript Highlighted.”*

Below, we provide a point-by-point response to each comment.

Thank you once again for your valuable guidance and support.

Ayto A. Negash, MD.

Corresponding Author

**Editorial Comments’ and Responses**

**Journal Requirements:**

1. We have amended your Competing Interest statement to comply with journal style. We kindly ask that you double check the statement and let us know if anything is incorrect.

**Response**: We have reviewed and updated the Competing Interests statement based on PLOS guidelines to ensure full compliance. The statement accurately reflects that the authors have no financial or non-financial competing interests, and it has been included in the manuscript.

**2.** Please provide separate figure files in .tif or .eps format.  **Repose:** We have prepared and submitted separate figure file in the requested formats. The figure has been provided as a high-resolution **.tiff** file, in accordance with the PLOS figure guidelines.

3. Your manuscript is missing the following sections: Discussion. Please ensure these are present, and in the correct order, and that any references to subheadings in your main text are correct.

**Response:** The Discussion section Was already included in the manuscript. All subheadings are presented in the correct order, and references to sections in the main text have been updated accordingly.

4. We have noticed that you have uploaded Supporting Information files, but you have not included a list of legends. Please add a full list of legends for your Supporting Information files after the references list.

**Response**: We thank the editor for pointing this out. A full list of legends for all Supporting Information files has now been added **after the References section** in the revised manuscript, in accordance with the journal’s guidelines.

5.If the reviewer comments include a recommendation to cite specific previously published works, please review and evaluate these publications to determine whether they are relevant and should be cited. There is no requirement to cite these works unless the editor has indicated otherwise.

**Response:** No reviewer requested the addition of a specific reference**.**

**Reviewers' comments:**

**Review Comments to the Author and Response**

**Reviewer #1:**

1. The data were collected between April- June 2021, which is almost four years ago. Although the authors do not dismiss this as a cross-sectional study, there is little information on the impact of the pandemic phase of data collection (post-initial wave, pre-vaccination in Ethiopia) on the generalizability. The discussion must be clear:
• What is the stage of the pandemic in this?
• The way the findings could vary in other phases of the pandemic.
• The applicability of these results to mental health assistance following the pandemic.

**Response:** We thank the reviewer for noting the importance of contextualizing the data collection period. The study was conducted during the early post–first-wave phase of COVID-19 in Ethiopia (April–June 2021), when case numbers were high and vaccination coverage was minimal. This timing likely influenced psychological distress levels due to fear of infection, uncertainty, and disruptions in routine healthcare. We acknowledge that findings may differ in later pandemic phases, such as after widespread vaccination or improved health-system stability. Nonetheless, these results provide valuable insights into mental health vulnerabilities during acute public-health crises and remain relevant for informing post-pandemic mental health support strategies. We have clarified this in methodology and discussion section.

2. The criterion of the exclusion of critically ill patients or patients with acute exacerbation can be a great source of selection bias. Psychological distress may be the greatest in patients who have acute exacerbation's. The authors should:
• Measure the number of patients who were not included for this reason.
• Talk about how this omission may result in the underestimation of prevalence.
• This is one of the significant weaknesses in generalizability.

**Response:** We appreciate the reviewer’s comment regarding the potential for selection bias due to the exclusion of critically ill patients or those with acute exacerbations. We acknowledge that psychological distress may be highest in this group, and their exclusion may indeed result in underestimation of prevalence. We have now clarified in the Limitations section that these patients were excluded because acute medical events themselves are significant stressors, making it difficult to attribute psychological distress specifically to the COVID-19 pandemic. We also recognize that this exclusion may affect the generalizability of our findings and have explicitly noted this as a limitation.

3. Social desirability bias is cited as one of the possible limitations by the authors, but it is not fully dealt with. Considering the nature of face-to-face interviews and the culture:
• What did interviewers do to reduce this bias?
• Did it contain validation questions?
• Did the authors ever think of using anonymous self-administered questionnaires?
**Response:** Data collection relied on interviewer-administered questionnaires. This approach was intentionally chosen because many of our chronic-disease patients are older adults with limited literacy, making self-administered questionnaires infeasible. Previous studies in Ethiopia have shown that a substantial proportion of older adults and chronic patients have low literacy or no formal education , and national surveys such as the Ethiopian DHS rely exclusively on interviewer administration for this reason. While interviewer administration ensured comprehension and inclusion, it may also have increased the risk of social-desirability bias, potentially leading to underreporting of psychological symptoms
4. The paper gives 46.5% as having had multiple comorbidities, but Table 3 presents a lot of patients with their conditions overlapping. Please clarify:
• What was the method of counting multiple comorbidities (e.g., a patient with hypertension, diabetes, and heart disease = 3 conditions)?
• What was the range, median number of comorbidities?
• Was it that some of the combinations were more closely linked with psychological distress?

**Response:** We thank the reviewer for this important comment. In our study, multimorbidity was defined as the presence of **two or more chronic medical conditions**, with each condition counted separately (e.g., a patient with hypertension, diabetes mellitus, and chronic kidney disease was counted as having three comorbidities). The number of comorbidities per patient ranged from **1 to 4**, with a **median of 1**. Overall, **198 participants (46.5%)** had multimorbidity.

We observed that certain overlapping conditions, particularly **hypertension and diabetes mellitus,** were the most common combination and appeared to be associated with higher levels of psychological distress, likely reflecting the increased burden of disease management and perceived vulnerability to severe COVID-19 outcomes. The Methods, Results, and Discussion sections have been revised accordingly to clarify these points.

5. a) Multivariate Model Variable Selection: The authors selected p<0.25 in bivariate analysis to select the variables. Although this method is common, it might have irrelevant variables. Could you explain why this threshold was chosen, and this should be analyzed by sensitivity analysis with p<0.10.
**Response:** We used p < 0.25 in bivariate analysis as a threshold to ensure that potential predictors with moderate associations were not excluded prematurely, which is a widely recommended approach in logistic regression modeling. Additionally, we performed a sensitivity analysis using p < 0.10 to confirm that the selected variables remained consistent, and results were robust.

These clarifications are now included in the Methods and Results sections.

b) Multicollinearity: The authors state that they have verified the presence of multicollinearity, although they do not provide the values of VIF. Since there is a likelihood of correlation between age, duration of illness, and age at onset, please provide:
• Final models of VIFs of all variables.
• Continuous variable correlation matrix.

**Response:** Thank you for this important comment. In the revised manuscript, we have now included a comprehensive multicollinearity assessment. Variance Inflation Factors (VIF) and Pearson correlation coefficients were computed for all continuous variables. Age at onset of illness showed severe multicollinearity with both age and duration of illness (VIFs: 57.1, 62.8, and 19.5; correlations r = 0.986 and r = 0.965), and therefore this variable was excluded from the final multivariable models.

The complete VIF table and the full continuous-variable correlation matrix have been provided in Supplemental File 1 legend as Multicollinearity assessment and correlation matrix of continuous variables, and the main findings are summarized in the Methods and Results sections of the revised manuscript.

c) Model Fit Statistics No goodness-of-fit statistics are provided on the logistic regression models (e.g., Hosmer-Lemeshow test, AIC, pseudo-R 2). These should be included.
**Response:** Thank you for this valuable comment. We have now incorporated the full set of recommended model fit statistics for all regression models.

- For the two binary logistic regression models (depression and anxiety):
  • The *Hosmer–Lemeshow goodness-of-fit test* indicated adequate model fit (p > 0.05).
  • *Pseudo-R² (Nagelkerke R²)* values have been added to quantify the explained variance.
  • *AIC values* were used to compare alternative model specifications.
- For the ordinal logistic regression model (perceived stress):
  • The *proportional odds assumption* was assessed using the *Test of Parallel Lines*, which indicated that the model was appropriate (p > 0.05).
  • Additional model fit statistics, including *Pseudo-R² values* and *AIC*, have also been added.

All model fit results have been incorporated into the Results section, and the corresponding analytic procedures are described clearly in the Data Analysis subsection.

d) Ordinal Logistic Regression: Did proportional odds assume low/moderate/high levels of perceived stress? The authors state that they do the test of parallel lines, but they do not give the results.
 **Response:** We have revised the Data Analysis section to comprehensively describe all diagnostic procedures conducted prior to model estimation.

- For the ordinal logistic regression model (perceived stress):
  The proportional odds assumption was evaluated using the Test of Parallel Lines, which indicated that the assumption was satisfied (*p* > 0.05).

These diagnostic procedures have been fully described in the revised Methods and result section.

6. The fact that stress was reported higher among physically active patients (AOR=1.9, p=0.005) is counterintuitive and should be given some more consideration. The concise explanation of the authors (an increased risk of exposure) is speculative. Consider:
• Is this reverse causation (stressed patients working out to get better)?
• Were there variations in kinds of physical activity?
• This observation is contrary to the literature, and it requires more in-depth investigation.
**Response:** We thank the reviewer for highlighting this important point. In response, we have revised the Discussion section to provide a more nuanced explanation of this unexpected finding. First, it is possible that higher stress levels led some patients to increase physical activity intentionally as a coping strategy, suggesting a potential reverse causation effect. Second, our data did not differentiate between types, intensity, or context of physical activity (e.g., occupational versus recreational), which may have influenced perceived stress levels. Third, certain forms of physical activity, particularly outdoor or occupational exercise, may have increased perceived risk of COVID-19 infection, thereby contributing to higher stress. Although most previous studies report that physical activity reduces stress, our findings highlight a potentially unique dynamic among patients with chronic diseases during a pandemic. We note that this counterintuitive observation warrants further investigation to clarify the underlying mechanisms and contextual factors. These explanations have been integrated into a detailed paragraph in the revised Discussion, placed after the section discussing comorbidities and before the overall summary paragraph to maintain logical flow, and the changes are highlighted in the submitted manuscript.

7. Figure 1 indicates k=7, though it is referred to as systematic random sampling. Please explain whether k was different by clinic or not.
• The rate of respondents was very good (97.4), and what about the 11 non-responders? Were they systematically dissimilar?

**Response**: We estimated the average patient load with comorbidities at the two selected tertiary hospital OPDs over the three months preceding data collection. The sample size was proportionally allocated based on these patient loads between the two hospitals and then further proportionally distributed among the six selected clinics within each hospital. The sampling interval $\text{k}$ was calculated by dividing the combined average patient load of both hospitals by the total sample size, resulting in a single common $\text{k}$ value applied consistently across all clinics and hospitals. Eligible patients were then selected using systematic random sampling by enrolling every $\text{k}$th patient, ensuring proportional and unbiased representation across the study sites.

Of the 11 non-responders, we reviewed clinic records for age, sex, and type of chronic disease. No significant differences were observed compared to the 397 participants, suggesting that non-response was unlikely to systematically bias the results. This clarity is now incorporated in the revised manuscript of methodology section

8. The PSS-10-C is said to be pandemic-related, but we should verify [34, 35]. Was this particularly confirmed in Ethiopia regarding COVID-19?

**Response:** We thank the reviewer for this important point. We clarify the following:

- The PSS-10-C is a pandemic-specific adaptation of the Perceived Stress Scale, designed to assess stress specifically related to COVID-19.
- While the PSS-10-C has been used internationally with acceptable reliability, no published psychometric validation studies have been conducted in Ethiopia.
- The standard PSS-10 has been validated in Ethiopian populations (e.g., Amharic versions among university students) and demonstrates acceptable psychometric properties.
- We acknowledge this as a limitation and highlight the need for future research to validate the PSS-10-C in Ethiopia

9. The Cronbach alpha of PSS-10-C is not high but satisfactory (0.783). Bad item-total correlations?

**Response:** We thank the reviewer for this observation. The Cronbach alpha of 0.783 indicates satisfactory internal consistency for the PSS-10-C in our sample. We examined item–total correlations for all 10 items, and each item demonstrated acceptable values (>0.30). No items showed poor performance, and therefore all items were retained in the analysis.

a) Table 1 & 3 (this should be Table 2): The numbering of the table seems wrong. The second table is actually Table 3 on page 15.

**Response:** We re-reviewed the manuscript carefully, including both the main text and the tables. We confirm that the tables are numbered and cited correctly in the current version

All table numbers in the text match the table sequence in the Results section. It is possible that the confusion may have arisen from one of the following:

- Formatting differences between the reviewer’s PDF version and the original Word document (e.g., page breaks shifting table placement).
- Automatic PDF conversion in the submission portal may have rearranged page order

To avoid any future confusion, we rechecked the alignment of table numbers, in-text citations, and captions and ensured consistent formatting across the entire revised manuscript.

The numbering is now correct and consistent throughout the document.

b) Age Categories: The age categories (18-38, 39-50, 51-62, >62) do not seem to be arbitrary. What was the rationale? It is better to use common age groups (e.g., 10-year intervals) to make the study more comparable to others.
 **Response:** Thank you for this insightful comment. We initially explored using standard 10-year age intervals; however, during data inspection we found that some age groups had very small numbers of participants, particularly at the younger and older ends of the distribution. This resulted in sparse cells, which would have limited the statistical power and stability of the regression model.

To address this and ensure adequate sample size within each category, we merged adjacent age bands to produce more balanced and analytically meaningful groups. The final categories (18–38, 39–50, 51–62, >62) were therefore derived based on:

1. Ensuring sufficient cell counts for reliable parameter estimation
2. Maintaining a logical progression of age groups
3. Avoiding empty or near-empty categories that compromise model fit

We have added a clarifying sentence in the Methods section to explain this rationale.

c) Missing Data: No discussion of missing data. Are there any declined responses? How were they handled?

**Response:** We appreciate the reviewer’s observation. Questionnaires were checked for completeness during data collection, resulting in no missing values for key outcome variables (PSS-10-C, HADS, Oslo-3). Minimal missing responses (<1%) occurred among socio-demographic variables and were handled using complete-case analysis. This has now been clearly stated in the manuscript.

10. The abstract indicates severe stress (45.5%), depression (39.7%), and anxiety (26.5%).
**Response:** The percentages reported in the abstract reflect the measured outcomes: severe stress is reported separately (45.5%), while depression (39.7%) and anxiety (26.5%) correspond to the combined prevalence of borderline and abnormal cases as measured by the HADS.

- We have revised the abstract to explicitly indicate that depression and anxiety prevalence includes both borderline and abnormal levels, ensuring clarity for readers.

11. Table 5 (Table 12 in the list), however, displays the depression and anxiety as yes/no, but also by level.

**Response:** We clarified that table 5 (Table 12 in the list) now properly labelled as Table 3 and we add the table footnote that the “Yes” category in Table 3 corresponds to participants with either borderline or abnormal symptoms.

- The table now reports both overall prevalence (borderline + abnormal) and the breakdown by level (borderline and abnormal separately), maintaining transparency while matching the abstract and results text.

12. In their reporting of depression at 39.7, is this combined with borderline (11%) + abnormal (28.6)? Please clarify throughout.
**Response:** Yes, the reported prevalence of 39.7% for depression includes both borderline (11%) and abnormal (28.6%) cases.

- We have clarified this explicitly in the Results section: “The prevalence of depression (39.7%) and anxiety (26.5%) reflects the combined proportion of participants with borderline and abnormal levels.”
- Table 3 and the abstract have also been updated to ensure consistent reporting of this combined prevalence.

13. a) Comparison with Pre-pandemic Data: You compare with a pre-pandemic Ethiopian study [42], but it would be nice to see more pre-pandemic Ethiopian baseline data in the discussion, provided that it exists.

**Response:** We have now expanded our discussion by incorporating additional pre-pandemic Ethiopian baseline data on depression, anxiety, and stress among patients with chronic illnesses. We added comparisons from studies conducted before COVID-19 in different regions of Ethiopia where available. This revision provides a clearer benchmark for understanding how the pandemic altered psychological outcomes in this population. We have revised the discussion accordingly

b) Clinical Implications: The prevalence and determinants have received much discussion, although little guidance is given on:
• Which particular interventions can be performed in the conditions of resource limitation?
• What is the way of incorporating mental health screening into chronic disease clinics?
• What are the implications of the health system?
**Response:** We have expanded the clinical implications section to include specific, feasible mental health interventions suitable for resource-limited settings, recommendations for integrating mental health screening into routine chronic disease care, and broader implications for the Ethiopian health system. These additions strengthen the practical relevance of our findings. The revised text appears at the end of the discussion.

c) Contradictory Results: In the age and stress discussion, you have support of younger adults experiencing higher stress (Iran) and older adults experiencing higher stress (Ethiopia, Turkey). The discussion will be enhanced by increasingly questioning why you have found a correlation with certain studies rather than others.
**Response:** Thank you for this insightful comment. We have now expanded the discussion to explicitly address the contradictory findings regarding age and psychological distress. A new paragraph has been added to explain why our results align with some studies but differ from others, emphasizing contextual, socio-economic, methodological, and cultural factors that may account for these variations. This addition strengthens the interpretation of our findings and clarifies the role of broader contextual influences.

14. The limitations are properly recognized; however, the limitations are to be added with:
    • Absence of pre-pandemic baseline data for the same patients.
    • Failure to determine the status of COVID-19 infection or loss of family and loved ones.
    • None of the past mental health history (not within treatment).
    • One-point assessment in a dynamic pandemic.
    • Page 4: "2740% of patients with chronic obstructive lung disease (COPD)- initial mention, COPD needs to be spelled out completely, followed by the abbreviation.
    • Page 5: the lifestyle modifications should be lifestyle modifications.
    • Page 6: please spell out (Gregorian Calendar?) GC on June 30, 2021GC
    • Unstable abbreviations: OPD vs outpatient department.
    **Response:** We have expanded the limitations section to acknowledge that we lacked pre-pandemic baseline mental health data for the same individuals, which restricts our ability to assess individual-level changes. We also added that participants’ COVID-19 infection status and experiences of losing loved ones were not assessed and may have influenced mental health outcomes. Although individuals with known mental illness were excluded, we clarified that reliance on self-report and medical records may not fully capture undiagnosed past mental health problems. Additionally, we noted that the cross-sectional, one-time assessment conducted during a dynamic pandemic limits the ability to observe changes over time, and we recommend longitudinal studies for future research. Finally, all minor issues related to abbreviations, spelling, and formatting (COPD, OPD/outpatient department, “lifestyle modifications,” and GC notation) have been corrected in the revised manuscript
15. You identified scores as poor (3-8), moderate (9-11), and strong (12-14). Nonetheless, only 3 items will be rated on a scale of 1-5, so it should be 15 instead of 14. Please confirm that your scoring system is equivalent to your references [32, 33]. The discussed methods are the ways to evaluate environmental, socio-economic, and health-related issues related to COVID-19, although these data are not provided in the results. Were these collected? In that case, they would be a useful context.
    **Response:** We thank the reviewer for raising this point. The **OSLO Social Support Scale (OSSS-3)** consists of three items, each with different scoring ranges. While some items have a maximum score of 5, the first item (“number of close confidants”) has a maximum score of 4 in the original OSSS-3, resulting in a total possible score ranging from **3 to 14**, as reported in references [32, 33]. Accordingly, the categorization of social support as **poor (3–8), moderate (9–11), and strong (12–14)** is consistent with the original OSSS-3 scoring system and aligns with the cited references. Regarding environmental, socio-economic, and health-related factors related to COVID-19, these variables were collected to provide contextual information but were not included in the results because the primary focus was on psychological outcomes (stress, anxiety, depression). We have clarified in the methods and discussion sections that these factors offer important context for interpreting the findings, ensuring transparency and completeness.

16. You have applied 55.7% prevalence in [28] and at the same time 22.8% in [27]. Why choose 55.7%? A smaller sample would be required to make a more conservative estimate. This move has to be explained.
**Response:** We thank the reviewer for highlighting this point. We selected the 55.7% prevalence reported in [28] for the sample size calculation because it represents the highest reported prevalence of psychological distress among similar populations in Ethiopia, ensuring that our study would be adequately powered to detect outcomes even in subgroups at higher risk. Using a smaller prevalence, such as 22.8% from [27], would have led to a smaller sample size and increased the risk of underpowering the study. Therefore, choosing the higher prevalence was a conservative approach to ensure sufficient sample size, improve precision, and account for potential nonresponse or missing data. We have clarified this rationale in the Methods section.
**Essential Revisions:**
• The numbering of tables is correct.
• Explain the count of multiple comorbidities and give descriptive statistics.
• All regression model fit statistics Report models.
• Give VIF values to show that there is no multicollinearity.
• Be more expansive on the context of the time and the generalizability.
• Discuss the physical activity paradox in more detail.
• Include clinical, health system implications in the discussion.
• Be more specific on how selection bias can be a problem.
• Add information on COVID-19-related stressors in case they were gathered.
• Take into consideration stratified analysis based on the type of chronic condition.
**Response:** We thank the reviewers for their thoughtful suggestions and have undertaken the following revisions to strengthen the manuscript. We confirmed that all table numbering is correct. Descriptive statistics for multiple comorbidities have been added, including counts and ranges per patient, to clarify the distribution of chronic conditions. All regression model fit statistics have now been reported, and variance inflation factor (VIF) values are included to demonstrate that multicollinearity does not affect the models. We have expanded the discussion on the context of data collection, highlighting the timing of the study during the dynamic COVID-19 pandemic and the limitations this imposes on generalizability, particularly to rural populations and lower-level health facilities. The “physical activity paradox” observed, where higher reported activity was associated with increased stress, has been discussed in greater detail, including potential reverse causation and exposure-related explanations. Clinical and health system implications have been incorporated, emphasizing evidence-based, low-cost interventions, integration of mental health screening into chronic disease clinics, and policy recommendations for integrated care models. Selection bias is now discussed more specifically, including the potential underestimation of psychological distress due to exclusion of critically ill patients, those with pre-existing mental health conditions, and individuals who avoided care. We also clarified that COVID-19-related stressors, such as infection status or loss of loved ones, were collected as contextual information but were not included in the primary results. Finally, highlighting differences in psychological outcomes among specific patient subgroups. These revisions collectively improve the transparency, rigor, and interpretability of our study.

**Reviewer #2: General Comments**
This study addresses an important topic regarding the mental health impact of COVID-19 on patients with chronic comorbidities in Ethiopia. The research is timely and contributes to understanding vulnerable populations during the pandemic. However, there are several limitations and areas requiring clarification or improvement.

Major Limitations and Concerns

1. Temporal Validity and Relevance
   Data collection period (April-June 2021) represents a specific pandemic phase that may not reflect current or evolving mental health impacts
   The cross-sectional design captures only a snapshot during one pandemic wave, limiting understanding of how psychological distress evolved over time.
   Consider acknowledging that findings may be specific to that pandemic period and discussing how mental health trajectories may have changed since
   **Response:** We revised the Discussion in the Limitations section to explicitly contextualize the study period as the early post–first-wave phase of COVID-19 in Ethiopia (April–June 2021), when vaccination had just begun and most participants were unvaccinated. We also clarified that the cross-sectional design captures mental health outcomes during a specific pandemic phase and may not reflect later trajectories. All changes and clarifications have been made in the revised manuscript.

2. Selection Bias and Generalizability
Exclusion of patients with pre-existing mental health conditions creates significant selection bias. These patients likely experienced the most severe psychological impact during COVID-19, yet they were systematically excluded
This exclusion means your prevalence estimates are likely underestimates of the true burden
The sample includes only patients who continued attending outpatient clinics. Patients who stopped seeking care due to fear, financial constraints, or severe psychological distress were missed
Both hospitals are tertiary referral centers in the capital city, limiting generalizability to rural populations or secondary healthcare settings

**Response:** We thank the reviewer for this important observation. We agree that excluding patients with known pre-existing mental health conditions may have resulted in underestimation of the true burden of psychological distress during the COVID-19 pandemic. This exclusion was applied to reduce confounding between pre-existing psychiatric disorders and pandemic-related psychological outcomes; however, we acknowledge that this decision introduces selection bias by systematically omitting individuals who may have experienced more severe distress.

We have revised the Limitations section to explicitly state that prevalence estimates may represent conservative estimates of the true psychological burden among patients with chronic diseases.

Additionally, we acknowledge that the sample included only patients who continued attending outpatient clinics during the pandemic. Individuals who avoided care due to fear of infection, financial hardship, or severe psychological distress were likely missed, further limiting representativeness. We have clarified this limitation in the Limitations section

Finally, we have expanded the discussion of generalizability to note that the study was conducted in two tertiary referral hospitals in Addis Ababa, which may limit applicability to rural populations and patients receiving care at secondary or primary healthcare facilities. These points are now more clearly articulated in the revised manuscript.

3. Social Desirability Bias
You acknowledge this limitation but do not discuss its potential magnitude
Face-to-face interviews may have led participants to under report psychological symptoms, particularly in a culture where mental health stigma exists
Self-administered questionnaires might have reduced this bias
**Response:** We agree with the reviewer that social desirability bias may have influenced the findings, particularly given the use of face-to-face interviewer-administered questionnaires and the presence of mental health stigma in the study context. Although interviewer administration was necessary due to low literacy among many participants, this approach may have led to under reporting of psychological symptoms.

In response, we have expanded the Limitations section to explicitly discuss the likely direction and potential magnitude of this bias, noting that prevalence estimates may be underestimated. We acknowledge that self-administered questionnaires might have reduced this bias but were not feasible in this setting.

4. Lack of Comparison Group
No pre-pandemic baseline data for the same population
No concurrent control group (e.g., patients without chronic conditions or general population)
This makes it difficult to attribute findings specifically to the pandemic versus chronic illness burden alone
**Response:** As noted, pre-pandemic mental health data for the same participants were not available, and no concurrent control group (e.g., individuals without chronic conditions or the general population) was included. This limits our ability to disentangle the psychological impact of COVID-19 from the underlying burden of chronic illness.

To address this concern, we have strengthened the Limitations section to explicitly state that causal attribution to the pandemic alone cannot be made. We also clarified in the Discussion section that comparisons with pre-pandemic Ethiopian studies were used only for contextual interpretation rather than causal inference.

5. Causality and Temporal Relationships
The cross-sectional design precludes establishing whether chronic conditions preceded or followed mental health symptoms
Cannot determine if COVID-19 stress caused the observed mental health outcomes or if pre-existing distress was simply measured during the pandemic
**Response:** We fully agree with the reviewer. The cross-sectional design does not allow determination of whether psychological symptoms preceded or followed the onset of chronic illness, nor whether COVID-19-related stress caused the observed outcomes or merely coincided with them.

We have revised the Limitations section to emphasize that temporal and causal relationships cannot be established and that findings should be interpreted as associations observed during a specific phase of the pandemic. We further note in the Discussion section that longitudinal studies are required to clarify directionality and causal mechanisms.

**Methodological Concerns Requiring Clarification**
6. Sampling and Response Rate
While you report a 97.4% response rate (426/437), this seems exceptionally high for voluntary participation in mental health research
Please clarify: Were patients approached consecutively? How many refused before the interview began?
The systematic sampling assumes all eligible patients attended during the study period—how did you handle missed appointments or irregular attendance patterns?
**Response**: We thank the reviewer for this comment. The requested clarifications regarding the high response rate, the recruitment procedure, and handling of missed or irregular attendees have now been incorporated directly into the Sampling Technique section of the manuscript. Specifically, we clarified that patients were approached systematically during scheduled outpatient visits with a random start in each clinic. Out of 437 eligible patients, 11 declined participations, resulting in a final sample of 426 (97.4% response rate). As the study was conducted only among patients attending chronic outpatient clinics, individuals who missed appointments or had irregular attendance were not included in the sampling frame. This approach was necessary because the study aimed to assess psychological outcomes among patients actively engaged in care, and patients not attending clinics during the study period were not accessible for recruitment.

7. Multiple Comorbidities and Disease Complexity
46.5% had multiple comorbidities, but the analysis doesn't account for specific comorbidity combinations
Different chronic disease combinations may have differential psychological impacts (e.g., cancer + CKD vs. hypertension + diabetes)
Disease severity and functional status were not assessed
**Response:** We thank the reviewer for this important comment. In our study, 46.5% of participants had more than one comorbid condition; however, the study was not powered to examine the psychological impact of specific comorbidity combinations. Although individual chronic diseases were common, the distribution of specific disease combinations resulted in small subgroup sizes that would have yielded unstable and unreliable estimates if modeled separately. Additionally, disease severity, stage of illness, and functional status were not assessed, all of which may influence psychological outcomes. We have now explicitly acknowledged these limitations in the manuscript and highlighted the need for future studies with larger sample sizes and more detailed clinical characterization to evaluate the combined and severity-specific effects of multiple chronic conditions on mental health outcomes.

**8. Measurement Issues**
**PSS-10-C (COVID-specific perceived stress):**
You report using PSS-10-C but provide limited validation information for the Ethiopian context
Was this tool translated and validated specifically for Amharic speakers?
The cut-off of ≥27 for "high stress" needs better justification, is this validated in your population?
**HADS:**
While validated in Ethiopian populations, most citations refer to HIV patients or adolescents—different from your chronic disease population
Cultural adaptation details are missing
**Oslo-3:**
Very brief measure (3 items) of a complex construct
May not capture pandemic-specific changes in social support networks
**Response:** We thank the reviewer for the insightful comments regarding the measurement tools.

**PSS-10-C:** The pandemic-related Perceived Stress Scale of COVID-19 (PSS-10-C) was translated into Amharic using standard forward-backward translation procedures and pilot-tested to ensure comprehension and cultural relevance among our study population. In this study, the scale demonstrated satisfactory internal consistency (Cronbach’s alpha = 0.783), and item–total correlations for all items were above 0.30, indicating that none of the items performed poorly or required removal. Although the cut-off of ≥27 for high stress has not been formally validated in Ethiopia, we adopted this threshold based on prior international literature and the observed distribution of scores in our population, acknowledging this as a limitation.

**HADS:** The Hospital Anxiety and Depression Scale (HADS) has been previously validated in Ethiopia; however, most existing studies focus on HIV patients or adolescents. While formal cultural adaptation for patients with chronic medical conditions was not performed, the scale has demonstrated robust psychometric properties across diverse Ethiopian populations, and its use in our study provides a practical and reliable assessment of anxiety and depression in this population

**Oslo-3:** We acknowledge that the Oslo-3 social support scale is a brief three-item instrument and may not fully capture pandemic-specific changes in social support networks. Despite this limitation, it has been widely used in Ethiopia and provides a valid, practical measure of general social support in the context of a larger survey.

Overall, we have highlighted these limitations in the Methods and Discussion sections and recommended that future studies conduct formal validation of these tools in the Ethiopian chronic disease population and explore more comprehensive measures of social support.
 **9. Statistical Analysis Concerns**
 **Reviewer comment:** Multicollinearity: You mention checking for it but don't report VIF values or tolerance statistics

**Response:** In the revised manuscript, we have now included a comprehensive multicollinearity assessment. Variance Inflation Factors (VIF) and Pearson correlation coefficients were computed for all continuous variables. Age at onset of illness showed severe multicollinearity with both age and duration of illness (VIFs: 57.1, 62.8, and 19.5; correlations r = 0.986 and r = 0.965), and therefore this variable was excluded from the final multivariable models. The complete VIF table and full continuous-variable correlation matrix are provided in Supplemental File 1, and the main findings are summarized in the Methods and Results sections.

**Reviewer comment:** Model fit statistics: No goodness-of-fit indices reported for logistic regression models

**Response:** We have now incorporated the full set of recommended model fit statistics for all regression models.

- Binary logistic regression models (depression and anxiety): The Hosmer–Lemeshow test indicated adequate model fit (p > 0.05), Nagelkerke pseudo-R² values were added to quantify explained variance, and AIC values were reported for model comparison.
- Ordinal logistic regression model (perceived stress): The proportional odds assumption was assessed using the Test of Parallel Lines (p > 0.05), and additional model fit statistics including pseudo-R² and AIC were included.
  All model fit results are now presented in the Results section, and corresponding analytic procedures are clearly described in the Methods section.

**Reviewer comment:** Multiple testing: With numerous comparisons, no correction for multiple testing is mentioned

**Response:** We acknowledge that multiple statistical comparisons were performed in this study without formal correction for multiple testing. This may increase the likelihood of Type I errors; therefore, the findings, particularly those approaching significance, should be interpreted with caution. Further research with larger samples and confirmatory analyses is recommended to validate these results.

**Reviewer comment:** Effect sizes: Only odds ratios presented, consider reporting standardized effect sizes for better interpretability

**Response:** We thank the reviewer for this suggestion. In the manuscript, we presented odds ratios (ORs) from logistic regression models to indicate the strength and direction of associations. While ORs provide a measure of effect, we acknowledge that reporting standardized effect sizes can enhance interpretability across variables with different scales.

10. **Missing Pandemic-Specific Variables:**

**Reviewer comment:**

- No assessment of COVID-19 infection history among participants or family members
- Vaccination status not captured (though this may predate widespread vaccination in Ethiopia)
- No measure of pandemic-related losses (income, employment, family deaths)
- Healthcare disruption not quantified (missed appointments, delayed treatments)

**Response:**
We thank the reviewer for highlighting these important aspects.

1. **COVID-19 infection and vaccination history:** The study was conducted between April and June 2021, during the early post–first-wave phase of the pandemic in Ethiopia, prior to widespread vaccination. Information on participants’ or their family members’ COVID-19 infection status was not systematically collected. We have acknowledged this as a limitation in the revised manuscript.
2. **Pandemic-related losses:** Although we did not collect detailed data on income loss, employment disruption, or family deaths, we recognize that these factors could meaningfully influence psychological outcomes. This limitation has now been explicitly noted.
3. **Healthcare disruption:** While the primary focus of our study was on patients attending chronic outpatient clinics, we did not quantify missed appointments or delays in treatment. We acknowledge that disruptions in healthcare access during the pandemic could have contributed to stress, anxiety, and depression, and this has been added to the Limitations section.

These clarifications have been incorporated into the revised manuscript to provide context for interpreting the psychological outcomes and to guide future research.

**Minor Limitations and Suggestions**

**11. Incomplete Characterization of Sample:**

**Reviewer comment:**

- Median household income or poverty indicators not reported
- Distance traveled to hospital not assessed (relevant for access and stress)
- Urban vs. rural origin within “outside Addis Ababa” not differentiated
- Specific chronic disease duration and severity not detailed

**Response:**
We thank the reviewer for this important comment.

- **Socioeconomic indicators:** Although we collected basic demographic and occupational data, detailed measures of household income or poverty status were not available. We have acknowledged this limitation in the revised manuscript.
- **Access to care:** Distance traveled to the hospital and urban versus rural residence within the “outside Addis Ababa” category were not systematically recorded. We recognize that these factors may influence stress levels and access to care, and this has been noted in the Limitations section.
- **Disease-specific characteristics:** Specific duration and severity of chronic diseases were not measured in this study, which may have influenced psychological outcomes. This limitation has now been explicitly mentioned in the manuscript.

These clarifications have been incorporated to provide context for interpreting the findings and to guide future studies that may include more detailed sociodemographic and clinical characterization.

**12. Physical Activity Paradox:**

**Reviewer comment:**

- Physically active patients had higher stress (AOR = 1.9), which is counterintuitive
- Possible reverse causation (anxious patients exercising more to cope)
- Physical activity assessment was binary (yes/no); intensity, duration, and type not captured

**Response:** The apparent association between physical activity and higher stress may reflect **reverse causation**, where patients experiencing higher stress engage in physical activity as a coping mechanism. As noted, physical activity was assessed using a binary measure in our study, without detailed information on type, intensity, or duration. This limitation reduces the precision of interpretation and may contribute to the unexpected association. We have acknowledged this issue in the revised manuscript and emphasized that future studies should collect detailed measures of physical activity and consider temporal relationships to clarify causality. These clarifications have been incorporated into the Discussion section to contextualize this finding.

**13. Data Quality Concerns:**

**Reviewer comment:**

- 2.8% substance use seems very low for a population under stress, possible underreporting
- 100% mask use is unusually high, suggesting social desirability bias
- Some variables (e.g., "being primary caregiver") are not clearly defined

**Response:**

- Substance use: We acknowledge that the reported prevalence (2.8%) may be underestimated due to social desirability bias and the reliance on self-report, particularly in a context where substance use is culturally sensitive. This limitation has been explicitly stated in the revised manuscript.
- Mask use: The observed 100% mask use likely reflects reporting bias, as participants may have responded according to perceived social norms rather than actual behavior. We have clarified this limitation in the Methods and Discussion sections.
- Variable definitions: The term “primary caregiver” has now been explicitly defined in the Methods section to indicate participants who are mainly responsible for the day-to-day care of a dependent household member.

These clarifications have been incorporated into the revised manuscript to improve transparency and interpretation of the data.

**14. Limited Exploration of Protective Factors:**

**Reviewer comment:**

- Study focuses primarily on risk factors
- Resilience, coping strategies, and positive adaptation not assessed
- Religious/spiritual support not evaluated despite mentioning religious festival restrictions as stressors

**Response:**
We acknowledge that our study primarily focused on identifying risk factors for psychological distress and did not include detailed measures of protective factors such as resilience, coping strategies, or religious/spiritual support.

- Resilience and coping strategies: While these factors are highly relevant, their assessment was beyond the scope of the current study. We have acknowledged this as a limitation in the revised Discussion section.
- **Religious/spiritual support:** Although we reported the potential impact of restrictions on religious festivals as contextual stressors, we did not systematically measure individual religious or spiritual support. This limitation is now clearly stated in the manuscript.

We emphasize in the revised Discussion that **future studies should include both risk and protective factors** to provide a more comprehensive understanding of psychological outcomes among patients with chronic conditions during pandemics.

15. **Language and Presentation:**

**Reviewer comment:**

- Some repetition between Results and Discussion sections
- Tables could be streamlined (Table 2 is very long)
- Statistical notation inconsistencies (CI presentation varies)

**Response:** In the revised manuscript, we have carefully addressed these issues as follows:

- **Repetition:** Redundant statements between the Results and Discussion sections have been removed or rephrased to improve clarity and flow.
- Table 2 has been streamlined by consolidating related variables, presenting percentages and counts more concisely, and reformatting the layout to fit on a single page for improved clarity while retaining all important information and variables.
- **Statistical notation:** All confidence intervals (CIs), p-values, and odds ratios have been standardized throughout the manuscript according to journal guidelines to ensure consistency and clarity. These revisions improve readability and enhance the presentation of our findings.

  **Recommendations for Authors**
  For Discussion Section:
  More explicitly address the selection bias from excluding patients with pre-existing mental health conditions and those who stopped attending clinics
  Discuss the physical activity paradox more thoroughly—this is a surprising finding that needs explanation
  Acknowledge that prevalence estimates are likely conservative due to multiple sources of underestimation
  Expand discussion of cultural context—how might Ethiopian cultural norms around mental health expression have influenced findings?
  Provide more context on healthcare system disruptions in Ethiopia during this period

**Response** For Discussion Section: In the revised manuscript, we have explicitly addressed potential selection bias, noting that excluding patients with pre-existing mental health conditions and those who discontinued clinic attendance may have led to underestimation of psychological outcomes. The physical activity paradox is discussed in more detail, considering possible reverse causation and the limitations of our binary activity measure (intensity, duration, and type not captured). We also acknowledge that prevalence estimates are likely conservative due to interviewer administration, stigma, and exclusion criteria. Additionally, we expanded the discussion of Ethiopian cultural norms, which may influence symptom reporting, and provided context on healthcare system disruptions during the pandemic, including reduced access to chronic care services and outpatient attendance. These revisions ensure the Discussion fully addresses the reviewer’s concerns and contextualizes our findings.

**For Limitations Section:**

The current limitations section is brief. Consider expanding to include:
Selection bias from excluding patients with mental health conditions
Inability to distinguish pandemic-specific effects from chronic illness burden
Lack of pre-pandemic baseline or control group
Potential social desirability bias in face-to-face interviews
Limited generalizability to rural populations and lower-level health facilities
Snapshot nature of cross-sectional data during one pandemic phase
Unmeasured confounders (disease severity, functional status, economic impact)
**Response for Limitations Section:** We have expanded the Limitations section to provide a more comprehensive overview. Our study may be affected by selection bias, as patients with pre-existing mental health conditions and those who discontinued outpatient visits were excluded, potentially underestimating psychological distress. The cross-sectional design and absence of a pre-pandemic baseline or control group limit our ability to distinguish pandemic-specific effects from the underlying burden of chronic illness. Face-to-face interviews may have introduced social desirability bias, particularly in a cultural context where mental health stigma persists. The findings may have limited generalizability to rural populations and patients attending lower-level health facilities. Additionally, the study provides only a snapshot during one pandemic phase, and several potential confounders, including disease severity, functional status, and economic impacts, were not measured. These factors should be considered when interpreting the results and highlight the need for longitudinal, contextually nuanced research.

For Future Research Recommendations:
Longitudinal studies tracking mental health trajectories
Inclusion of patients with pre-existing mental health conditions
Mixed-methods research incorporating qualitative data
Intervention studies testing psychosocial support programs
Comparison with pre-pandemic cohorts
Assessment of long-term mental health consequences
**Response for Future Research Recommendations:** We recommend for Future studies to employ longitudinal designs to track mental health trajectories over time and include patients with pre-existing mental health conditions to better capture the full spectrum of psychological burden. Mixed-methods approaches incorporating qualitative data could provide deeper insight into patients’ experiences and coping strategies. Intervention studies testing psychosocial support programs are warranted to identify effective strategies for mitigating stress, anxiety, and depression among chronic disease populations. Comparative analyses with pre-pandemic cohorts and assessments of the long-term mental health consequences of COVID-19 will further strengthen understanding of pandemic-related psychological impacts.

Specific Questions for Authors
Why were patients with pre-existing mental health conditions excluded? This seems to exclude the most vulnerable group.
**Response:** **Exclusion of patients with pre-existing mental health conditions:** Patients with known pre-existing mental health conditions were excluded to reduce confounding, as our primary objective was to assess pandemic-related psychological impacts among patients with chronic medical conditions. We acknowledge that this exclusion may have underestimated the overall psychological burden and discussed this as a limitation in the revised manuscript.

Can you provide validation data for PSS-10-C in the Ethiopian context?

**Response: Validation of PSS-10-C in the Ethiopian context:**
The PSS-10-C was translated into Amharic using forward-backward translation and pilot-tested for comprehension and cultural relevance. In this study, it demonstrated satisfactory internal consistency (Cronbach’s alpha = 0.783), with all items showing acceptable item–total correlations (>0.30). While formal validation studies in Ethiopia are still needed, these results support its reliability in our study population.

How did you handle patients with multiple comorbidities in the analysis? Were interaction effects explored?

**Response: Handling multiple comorbidities:** Multiple comorbidities were defined as two or more chronic medical conditions per patient. Due to the small number of participants with specific comorbidity combinations, interaction effects could not be robustly explored. Individual chronic conditions were analyzed for associations with psychological outcomes, and this limitation has been acknowledged in the revised manuscript.

What was the refusal rate before consent? The 97.4% response rate seems unusually high.

**Response of Response rate:** Thank you for this query. Of 437 eligible patients approached, 426 completed the questionnaire (response rate 97.4%). Eleven patients (2.6%) declined participation due to time constraints upon initial approach; there were no further refusals after consent explanation.This high rate aligns with our face-to-face, interviewer-administered design during routine clinic visits, where patients were a captive audience and the questionnaire imposed minimal burden. Rates >90–98% are common in similar clinic-based studies in Ethiopia and other low-resource settings.We have added the response rate calculation and refusal details to the Methods section for clarity.

Can you explain the counterintuitive finding regarding physical activity and increased stress?

**5. Physical activity and increased stress:**
The counterintuitive association of physical activity with higher stress (AOR=1.9) may reflect reverse causation, whereby patients experiencing higher stress engaged in physical activity as a coping mechanism. Additionally, physical activity was assessed as a simple yes/no variable; intensity, frequency, and type were not captured. This explanation has been added to the Discussion section.

Were any interventions offered to patients identified with severe depression, anxiety, or stress?

**6. Interventions for patients with severe symptoms:**
While no structured interventions were provided as part of the study, patients identified with severe depression, anxiety, or stress during data collection were referred to the mental health services for appropriate evaluation and care. This has been clarified in the revised Methods section.

**Figure Resubmissions:**

While revising your submission, we strongly recommend that you use PLOS’s NAAS tool ([https://ngplosjournals.pagemajik.ai/artanalysis](https://ngplosjournals.pagemajik.ai/artanalysis)) to test your figure files. NAAS can convert your figure files to the TIFF file type and meet basic requirements (such as print size, resolution), or provide you with a report on issues that do not meet our requirements and that NAAS cannot fix.

After uploading your figures to PLOS’s NAAS tool - [https://ngplosjournals.pagemajik.ai/artanalysis](https://ngplosjournals.pagemajik.ai/artanalysis), NAAS will process the files provided and display the results in the "Uploaded Files" section of the page as the processing is complete. If the uploaded figures meet our requirements (or NAAS is able to fix the files to meet our requirements), the figure will be marked as "fixed" above. If NAAS is unable to fix the files, a red "failed" label will appear above. When NAAS has confirmed that the figure files meet our requirements, please download the file via the download option, and include these NAAS processed figure files when submitting your revised manuscript.

**Response:** We have carefully revised the figure file as requested. The figure have been processed using PLOS’s NAAS tool [(https://ngplosjournals.pagemajik.ai/artanalysis)](https://ngplosjournals.pagemajik.ai/artanalysis) to ensure they meet the required specifications for file type (TIFF), resolution (300–600 dpi), print size, and file size limits. The tool successfully converted and optimized the file where needed, and we have uploaded the corrected versions with this revision.

We believe the revised figure now fully comply with PLOS technical requirements. Please let us know if any additional adjustments are needed.
